# Diversity and Genomic Characterization of a Novel Parvarchaeota Family in Acid Mine Drainage Sediments

**DOI:** 10.3389/fmicb.2020.612257

**Published:** 2020-12-21

**Authors:** Zhen-Hao Luo, Qi Li, Yan Lai, Hao Chen, Bin Liao, Li-nan Huang

**Affiliations:** School of Life Sciences, Sun Yat-sen University, Guangzhou, China

**Keywords:** novel family, Parvarchaeota, small genome, metabolic potentials, environmental adaptations

## Abstract

Recent genome-resolved metagenomic analyses of microbial communities from diverse environments have led to the discovery of many novel lineages that significantly expand the phylogenetic breadth of Archaea. Here, we report the genomic characterization of a new archaeal family based on five metagenome-assembled genomes retrieved from acid mine drainage sediments. Phylogenomic analyses placed these uncultivated archaea at the root of the candidate phylum Parvarchaeota, which expand this lesser-known phylum into two family levels. Genes involved in environmental adaptation and carbohydrate and protein utilization were identified in the ultra-small genomes (estimated size 0.53–0.76 Mb), indicating a survival strategy in this harsh environment (low pH and high heavy metal content). The detection of genes with homology to sulfocyanin suggested a potential involvement in iron cycling. Nevertheless, the absence of the ability to synthesize amino acids and nucleotides implies that these archaea may acquire these biomolecules from the environment or other community members. Applying evolutionary history analysis to Parvarchaeota suggested that members of the two families could broaden their niches by acquiring the potentials of utilizing different substrates. This study expands our knowledge of the diversity, metabolic capacity, and evolutionary history of the Parvarchaeota.

## Introduction

Advances in sequencing technology and computational approaches have enabled the reconstruction of microbial genomes directly from the environment. Such efforts have led to the discovery of major new lineages previously missing from the tree of life (Rinke et al., 2013; Baker et al., 2016; Baker et al., 2020). Extreme environments are pervasive landscapes on the planet. Previous molecular surveys have documented the predominance of uncultivated archaea under the most extreme environmental conditions (Huang et al., 2011; Podell et al., 2013). Recent genome-resolved metagenomics analyses of these low-diversity environments have retrieved near-complete or even closed genomes for these elusive taxa, resolving their metabolic functions and evolutionary histories and significantly expanding the archaeal phylogenetic tree (Dombrowski et al., 2018; Hug et al., 2016; Rinke et al., 2013; Baker et al., 2020).

Acid mine drainage (AMD) is a worldwide environmental problem, arising from the microbially-mediated oxidative dissolution of sulfide minerals (primarily pyrite, FeS2) during mining activities (Bond et al., 2000; Sánchez-Andrea et al., 2011). These extremely acidic, heavy metal-rich solutions pose a significant threat to the indigenous microorganisms. As an essential representative of extreme environments, AMD systems typically harbor low complexity microbial communities and have been subject to extensive metagenomic analyses (Denef et al., 2010; Huang et al., 2016). The Archaeal Richmond Mine Acidophilic Nanoorganisms (ARMAN), consisting of the candidate phyla Micrarchaeota and Parvarchaeota, were first discovered in the acidophilic biofilms in the Richmond Mine, Iron Mountain, United States (Baker et al., 2006, 2010). Several previous studies have applied genome-enabled metagenomics, comparative genomics, and cryogenic transmission electron microscope technology to provide initial insights into the metabolic capacities and potential ecological roles of these ultra-small archaea and demonstrate their physical interactions with Thermoplasmatales members in the community (Baker et al., 2010; Chen et al., 2018). However, the ARMAN metagenomic-assembled genomes (MAGs) analyzed in those studies are limited to a narrow phylogenetic breadth. For example, all currently available Parvarchaeota genome bins are affiliated with a single family-level group (Chen et al., 2018). Thus, to gain further insights into the physiology and ecology of these elusive archaea, reconstructing new MAGs from more diverse environmental samples and increasing genomic representation of these uncultivated phyla are necessary. Here, we report retrieval and comparative analysis of five new MAGs that may represent a novel Parvarchaeota family in AMD sediments.

In this study, five draft metagenome-assembled genomes (MAGs) representing a novel archaeal lineage were obtained from AMD sediment samples, whose relative abundance is relatively low (relative abundances less than 1%). Phylogenomic analysis showed that they were placed at the root of *Ca.* Parvarchaeota, an important clade in DPANN, which is characterized by small genome size and restricted metabolic repertoires. Newly recovered MAGs in this study have minuscule genome size (less than 0.57 Mb). Then, genome-resolved analysis revealed that they could utilize carbohydrates and proteins but might obtain certain metabolites from environments and/or community members because they lack many important metabolic pathways, including biosynthesis of amino acids and nucleotides. This study expands our understanding of the ecological roles of rare taxa and sheds light on the adaptation mechanisms of small-genome lineages in AMD environments.

## Materials and Methods

### Study Sites and Sampling

Acid mine drainage sediments were collected from three mine sites. (1) Fankou (FK) Pb/Zn mine (25° 2′ 56.5″ N, 113° 39′ 48.5″ E) located in Guangdong province of China. Two cores were sampled in the tailings impoundment in October 2017 as described previously (Gao et al., 2020). Only Core A was included in the current study due to the presence of the newly discovered Parvarchaeota lineage. The core was divided into six layers (A1–A6) based on their differences in colors and physical features. (2) Tongling (TL) pyrite mine (30° 94′5.41″ N, 117° 98′ 99.72″ E) located in Anhui Province of China. One AMD sediment sample was collected from a depth of 0–0.5 cm in August 2017. (3) Maanshan (MAS) iron mine (31° 67′72.16″ N, 118° 62′ 74.33″ E) located in Anhui province of China. Three AMD sediment samples (MAS1–MAS3) were collected in August 2017. All samples were collected into 50 ml sterile tubes and held in an icebox and transported within 24 h to the laboratory where they were stored at 4°C. DNA extraction was conducted typically within 48 h.

### Determination of Physicochemical Properties

The subsamples were air-dried and prepared for the determination of physicochemical properties as previously reported (Gao et al., 2020). In brief, pH and EC were measured using specific electrodes. The concentration of ferrous iron and ferric iron were determined by the 1,10-phenanthroline method at 530 nm. The concentration of sulfate (SO_4_^2–^) was measured with a BaSO4-based turbidimetric method. Heavy metals and total sulfur (TS) were measured by inductively coupled plasma optical emission spectrometry (ICP-OES, optima 2100DV; Perkin-Elmer, MA, United States). The contents of total organic carbon (TOC) and total phosphorus were determined with TOC-VCPH (Shimadzu, Columbia, MD, United States) and Smart Chem (Westco Scientific Instruments Inc., Brookfield, CT, United States) according to standard methods, respectively.

### DNA Extraction and Metagenomic Sequencing

DNA was extracted using FastDNA Spin kit (MP Biomedicals, Irvine, CA, United States) and purified with QIA quick Gel Extraction Kit (Qiagen, Chatsworth, CA, United States) according to manufacturer’s instructions. The quality and quantity of total community DNA were estimated using agarose gel electrophoresis and Qubit (Thermo Fisher Scientific, Australia). Finally, library preparation of DNA was conducted with NEBNext Ultra II DNA Prep Kit (New England Biolabs, Ipswich, MA, United States) and sequenced using an Illumina Hiseq2500 platform using a 150bp paired-end approach.

### Metagenomic Assembly, and Genome Binning, Annotation and Comparative Analysis

All raw data were filtered to remove duplicates using in-house Perl scripts, and low quality based/reads using Sickle v.1.33 (Joshi and Fass, 2011) with the following parameters: “-q 15 –l 50”. After that, high-quality reads were assembled (or co-assembled for the six FK sediments) using SPAdes v.3.9.0 (Bankevich et al., 2012) with the parameters: “-k21,33,55,77,9,127 –meta”. Reads from metagenomics datasets were mapped to the assembled scaffolds (length ≥ 2500 bp) to calculate scaffold coverage. For co-assembly quality datasets, reads from different datasets were mapped to the assembled scaffolds separately using BBMap v.36.77 with the same parameter set. To obtain more accurate binning results, scaffolds were binned using MetaBAT v2.12.1 (Kang et al., 2015), MaxBin v2.2.2 (Wu et al., 2015), Abawaca v1.00, and Concoct v0.4.0 (Alneberg et al., 2014) with default parameters to get the initial genome binning result, considering tetranucleotide frequencies, GC content, taxonomic affiliation, and abundance profiles of scaffolds. The binning result was then refined using DASTools v1.0 (Sieber et al., 2018) and manually curated using RefineM v0.0.24 (Parks et al., 2017). The MAGs sequences have been deposited to the NCBI with the project accession number: PRJNA666095. Due to the small size of Parvarchaeota MAGs, completeness of all MAGs was assessed using the occurrence of 54 archaeal marker proteins (Supplementary Table 1). Protein-coding sequences of all MAGs were predicted using Prodigal (Hyatt et al., 2010) v.2.6.3 with the “-p single” option. Then, they were assigned to functional orthologs of the KEGG Orthology (KO) database using DIAMOND (Buchfink et al., 2015) with E-value < 1e^–5^ to get a KO count table (Supplementary Table 2). The metabolic profiles of MAGs were manually verified based on the KEGG count table and online KEGG mapping tools.^1^ Carbohydrate-active enzymes (CAZy) and peptidases were identified using the CAZy database on the dbCAN webserver (Cantarel et al., 2009) and MEROPS (Rawlings et al., 2016) respectively. To predict signal peptides and localization of these enzymes (CAZy and peptidases), we applied two algorithms, SignalP-5.0 (Almagro Armenteros et al., 2019) and PSORTb v3.0 (-a option for archaea sequences, Yu et al., 2010). All available MAGs belonging to Parvarchaeota were obtained from NCBI and IMG-M database. Among them, three MAGs (*Ca.* Parvarchaeota archaeon Guaymas_33, *Ca.* Parvarchaeota archaeon CSSed11_243R1 and *Ca.* Parvarchaeota archaeon CSSed10_416R3) downloaded from NCBI were removed because they were located in “*Ca.* Pacearchaeota” phylum in phylogenetic analysis. Finally, 32 MAGs were taken into comparison, which information of published MAGs was summarized in Supplementary Table 3. Average amino acid identity (AAI) values of these MAGs were determined by CompareM v.0.0.24. For further comparative analysis, only MAGs with completeness >70% were selected. Orthologous groups (OGs) were identified by Orthofinder v.2.3.12 (Emms and Kelly, 2019), and gene gain and gene loss events were reconstructed using COUNT v.10.04 (Csûös, 2010) with Dollo parsimony.

### Phylogenetic Analyses

Sixteen ribosomal protein sequences (i.e., L2, L3, L4, L5, L6, L14, L15, L16, L18, L22, L24, S3, S8, S10, S17, and S19) were selected to generate phylogenetic trees of selected MAGs. Multiple sequence alignments (MSA) of the individual proteins were obtained using MUSCLE (Edgar, 2004) v3.8.31 with default parameters. Poorly aligned regions were filtered using trimAL (Capella-Gutiérrez et al., 2009) v1.4 with the parameters “-gt 0.95 –cons 50”. Maximum-likelihood phylogeny for these ribosomal proteins was inferred using the IQtree (Nguyen et al., 2014) v.1.6.10 with 1,000 ultra-rapid bootstraps (Hoang et al., 2018).

The 16S rRNA gene sequences were identified using SSU-ALIGN v.0.1.1.^2^ The alignment of 16S rRNA gene sequences was performed on SINA web interface^3^ (Pruesse et al., 2012). The protein sequences of sulfocyanin were retrieved by querying the RefSeq non-redundant proteins database (NR) using protein sequences from our MAGs and aligned with MUSCLE with default parameters. A concatenated set of 122 archaeal marker proteins were selected with GTDB-Tk (Parks et al., 2017). Then, trimAL was used to eliminate those columns with ≥95% gaps in 16S rRNA and sulfocyanin alignments. The trees were inferred using the IQtree as described above. All trees were uploaded to iTOL v.4 (Letunic and Bork, 2019) for visualization.

## Results and Discussion

### Novel Archaeal MAGs Recovered From AMD Sediments

**FIGURE 1:**
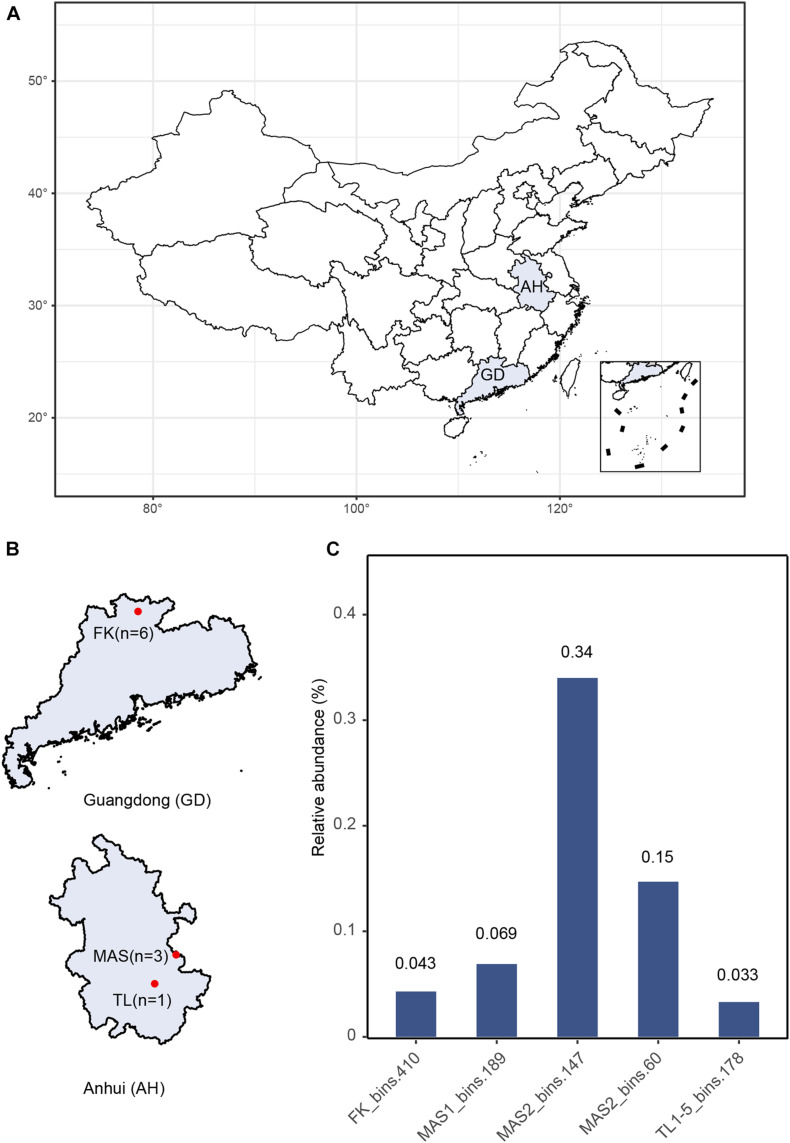
**(A)** Geographic locations of sampling sites. **(B)** Details of sampling sites and the number of samples. **(C)** Relative abundance of newly recovered MAGs, calculated by the abundance of the all scaffolds of each MAGs over the abundance of all scaffolds in corresponding samples.

Metagenomic sequencing was performed on ten sediment samples collected from three AMD environments (Figures 1A,B). The FK sediment core samples showed vertical gradients of physicochemical properties (Supplementary Table 4), with pH values shifting from extremely acidic at the surface layers to near neutral at the deeper layers. The other sediment samples were characterized by low pH values (2.5–2.8) (Supplementary Table 4) typical of AMD and associated environments (Rothschild and Mancinelli, 2001). After quality control, *de novo* assembly and genome binning, five novel archaeal MAGs were obtained, namely FK_bins.410, MAS1_bins.189, MAS2_bins.147 and MAS3_bins.60, and TL1-5_bins.178, respectively. These MAGs represent a rare group in the AMD environments with relative abundances of all MAGs <1% (Figure 1C and Supplementary Table 5). Estimated genome size ranges from 0.53 to 0.76 Mb (averagely, 0.65 Mb) and GC content ranges from 41 to 42%. On average, the MAGs encode 515 genes with an average gene length of 816 bp (Supplementary Table 5).

Based on the occurrence of 54 archaeal single-copy genes, genome completeness ranges from 63 to 80% (71% on average) (Supplementary Table 1).

### Phylogeny and Environmental Distribution of Novel Archaeal MAGs

**FIGURE 2:**
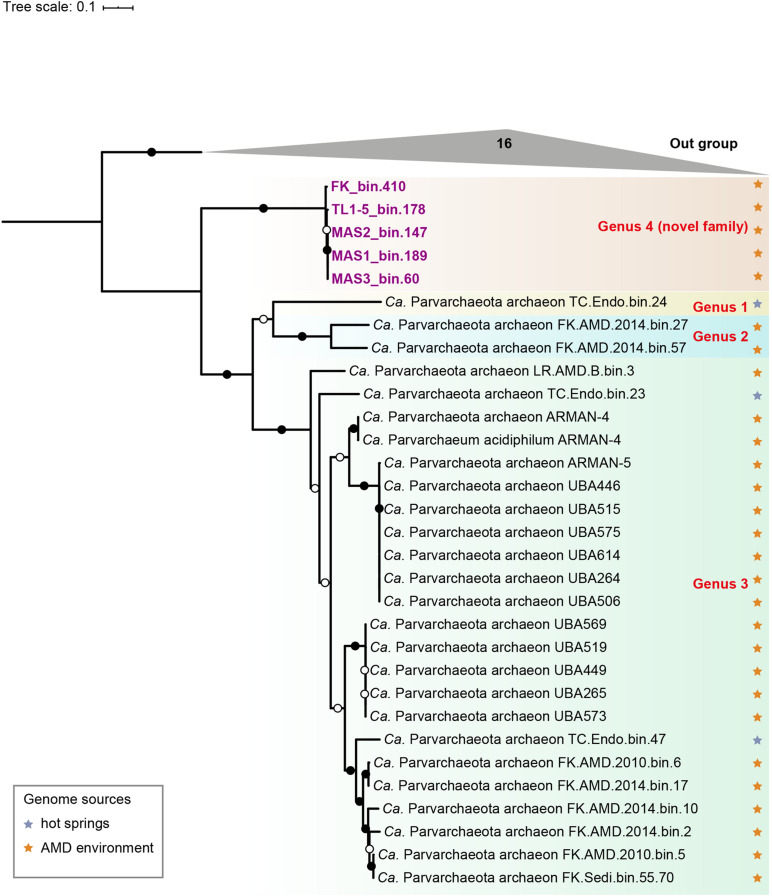
Phylogenetic tree of Parvarchaeota based on concatenation of 16 ribosomal proteins. Different clades are showed by boxes with diverse colors and MAGs newly obtained in this study are labeled with purple. Isolation sources of available Parvarchaeota MAGs are indicated by stars with different colors. Nodes with ultrafast bootstrap value ≥95% (50%) are indicated as solid (hollow) circles, and the scale bar at the bottom indicates 10% sequence divergence.

To resolve the phylogenetic affiliation of the new MAGs, a concatenated alignment of 16 ribosomal proteins was used to construct a maximum likelihood (ML) phylogenomic tree. The result showed that they formed a monophyletic group at the root of the candidate phylum Parvarchaeota (a clade within DPANN superphylum) with high bootstrap values (bootstrap support values = 100%, Figure 2). Additional analyses based on 122 concatenated archaea-specific protein markers and 16S rRNA gene further supported this result (Supplementary Figures 1, 2). It is assumed that Parvarchaeota are restricted in AMD and hot spring environments (Chen et al., 2018). Consistently, the phylogenetic tree based on 42 Parvarchaeota 16S rRNA gene sequences (three from the MAGs retrieved in the present study) confirmed that this phylum is likely limited to these two types of environment (Supplementary Figure 2). The AAI values of our MAGs to other Parvarchaeota MAGs (53–56%, Supplementary Figure 3 and **Table 6**) fell within the range of the threshold of the same family (Konstantinidis et al., 2017). However, 16S rRNA gene sequence identity (83–90%) between newly recovered MAGs and published Parvarchaeota MAGs (Supplementary Table 7), fits in the thresholds of family level (Yarza et al., 2014). Therefore, based on 16S rRNA gene sequence identity, phylogenetic and phylogenomic results, we suggested that these MAGs represent a novel family within Parvarchaeota. We proposed that our MAGs represent a novel family within Parvarchaeota and the name “*Candidatus* Acidifodinimicrobiaceae” fam. nov., and “*Candidatus* Acidifodinimicrobium mancum” gen. nov., sp. nov. with the genome serving as the type material deposited in GenBank under accession number GCA_015121965.1.

“*Candidatus* Acidifodinimicrobium” gen. nov.

A.ci.di.fo.di.ni.mi.cro’bi.um. L. masc. adj. *acidus* sour; L. fem. n. *fodina* a mine; N.L. neut. n. *microbium* a microbe; N.L. neut. n. *Acidifodinimicrobium* a microbe from an acidic mine environment.

Type species: *Ca.* Acidifodinimicrobium mancum.

“*Candidatus* Acidifodinimicrobium mancum” sp. nov.

*Candidatus* Acidifodinimicrobium mancum (man’cum. L. neut. adj. *mancum* maimed, defective, as the organism lacks the genes for amino acids and nucleotides synthesis.

The representative genome is 0.47 Mbp with a GC content of 42%. The estimated genome completeness is 80%, with approximately 0.97% contamination.

Type material: FK_bins.410^*T*^ (GCA_015121965.1), obtained from the metagenome assembly of AMD sediments from Fankou Mine.

“*Candidatus* Acidifodinimicrobiaceae” fam. nov.

A.ci.di.fo.di.ni.mi.cro.bi.a.ce’ae. N.L. neut. n. *Acidifodinimicrobium* a (*Candidatu*s) genus; -aceae ending to denote a family; N.L. fem. pl. n. *Acidifodinimicrobiaceae* the (*Candidatus*) *Acidifodinimicrobium* family).

**FIGURE 3:**
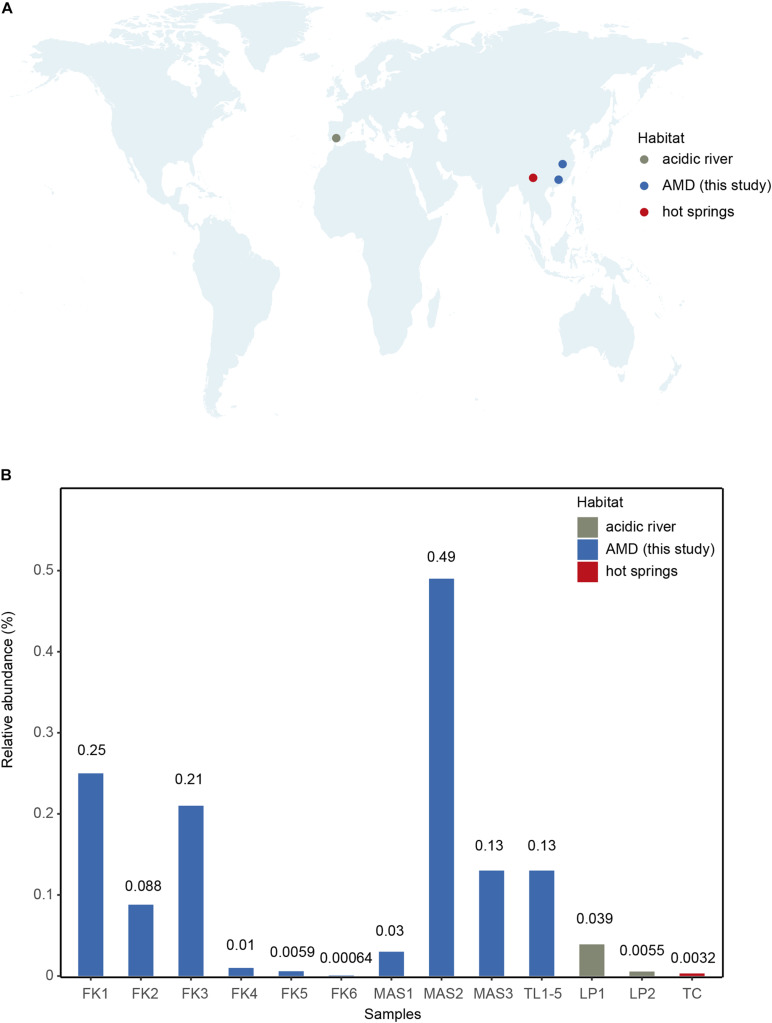
**(A)** Global distribution of the 16S rRNA genes of newly recovered Parvarchaeota MAGs based on IMNGS database with 97% sequence-identity threshold. The retrieved 16S rRNA sequences were plotted with different colors on the global map. **(B)** The relative abundance of newly recovered MAGs calculated by the number of 16S rRNA gene sequences over the whole size of corresponding samples.

As all currently available Parvarchaeota MAGs were classified as one family (Chen et al., 2018), the AMD sediment MAGs reported here further expanded the phylogenetic and genomic diversity of this lesser-known phylum. The environmental distribution of this new family was evaluated using the Integrated Microbial Next Generation Sequencing database and our database. The result showed that the new family occurs predominately in our samples and two acidic river samples from Spain (Figure 3 and Supplementary Table 8). Consistently, it represents a rare group as this family’s relative abundances were very low in these samples (average 0.11%, *n* = 13, Figure 3B and Supplementary Table 8). Besides, it was detected in a hot spring sample located in Tengchong county of Yunnan Province, China (Figure 3A), confirming that the distribution of Parvarchaeota is restricted in hot springs and AMD-related environments (Chen et al., 2018).

### Metabolic Potentials

**FIGURE 4:**
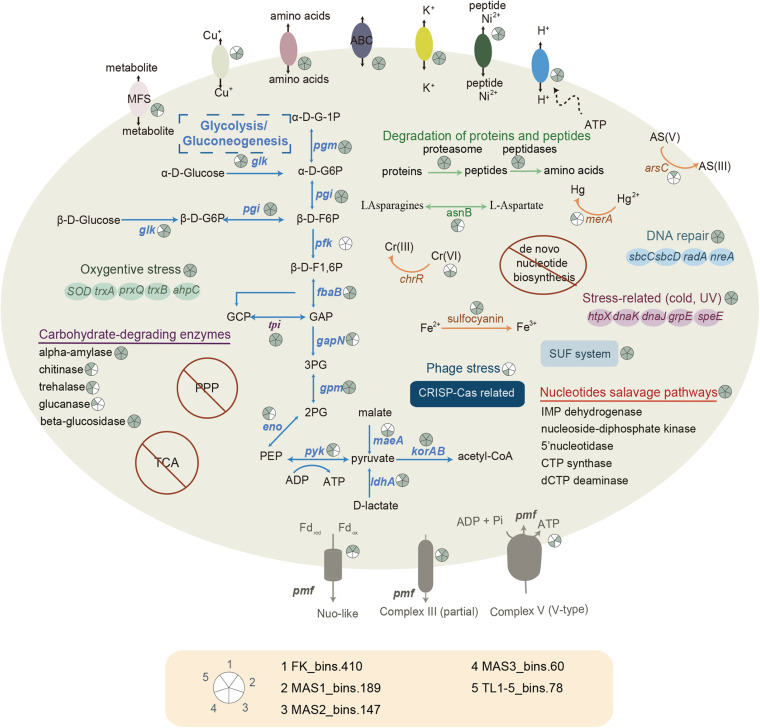
Reconstructed metabolic pathways of newly recovered Parvarchaeota MAGs. α-D-G, α-D-glucose; β-D-G, β-D-glucose; β-D-F, β-D-fructose; GCP, glycerone-P; GAP, glyceraldehyde-3P; 3PG, glycerate-3P; 2PG, glycerate-2P; PEP, phosphoenolpyruvate; Fd_*ox*_, oxidized ferredoxin; Fd_*red*_, reduced ferredoxin; pmf, proton motive force. For detailed on pathways, see Supplementary Table 9.

The metabolic repertoires of Parvarchaeota MAGs were investigated by comparisons of KEGG database. Principal coordinates analysis (PCoA) based on KOs was conducted to evaluate the relationship between newly recovered MAGs and published Parvarchaeota MAGs. It revealed that the functional profiles of these newly recovered MAGs were significantly different from others (PERMANOVA, *p* < 0.05, Supplementary Figure 4), which further confirmed that these recovered MAGs represented a novel lineage. Unlike other Parvarchaeota members (Chen et al., 2018), only a few transporters were detected in these recovered MAGs (Figure 4 and Supplementary Table 9), indicating a more limited functional versatility. However, genes encoding ABC-type peptide/nickel transporter systems were detected in these newly recovered MAGs but not reported in other Parvarchaeota members. Consistent with this, the presence of proteasome and a variety of peptidases in these MAGs (Supplementary Table 9), like serine (e.g., family S16 and S53), metallopeptidases (e.g., M48, M103), aspartic (e.g., A24), and other peptidases (e.g., archaeal-type methionyl aminopeptidase) suggested that they could scavenge peptides and proteins. Notably, the presence of N-terminal secretion signal peptides in some genes encoding serine peptidases indicated that they might be secreted to degrade peptides in the environments. Then, the occurrences of several enzymes (e.g., S-adenosylmethionine synthetase, asparagine synthase, and spermidine synthase) involved in amino acid metabolism could provide substrates and energy for these MAGs from the generated amino acids. Genes related to selenoamino acid metabolism were detected in this lineage. For example, *sufS* gene encoding for selenocysteine lyase, recycling selenium during the breakdown of selenoproteins and providing it for selenocysteine biosynthesis (Labunskyy et al., 2014) were detected in 4 MAGs. Selenocysteine is a component of glutathione peroxidase, thioredoxin reductase, glycine reductase and hydrogenases (Böck et al., 1991). Moreover, the occurrence of *yaaU* gene in four MAGs encoding MFS transporter (putative metabolite transporter protein) indicated that they could take up monosaccharides and polysaccharides, amino acids and peptides, vitamins, and coenzyme factors for survival.

To study the potential of this archaeal lineage to breakdown carbohydrates, we screened them for the occurrence of CAZymes (Supplementary Table 9). The presence of genes in the glycosyltransferase (GT) family indicated that they may be capable of biosynthesis of sugars (e.g., GT3, glycogen synthase) for storage. Besides, enzymes that belong to glycoside hydrolases (GH) were detected in all MAGs. Therefore, they might be able to degrade starch (GH57, alpha-amylase), chitin (GH18 and GH 19, chitinase), trehalose (GH37, α-α-trehalase), glucan (GH55, β-1,3-glucanase) and disaccharides (GH1, beta-glucosidase). Although none of them harbored a potential signal peptide at N-terminus, four genes (encoding alpha-amylase, GH57) distributed in four MAGs (MAS1_bin.189, MAS2_bin.147, MAS3_bin.60, and TL1-5_bin.178) were predicted to be extracellular, implying that some proteins may be involved in extracellular hydrolysis of starch. During the degradation of carbohydrates, glycolysis usually supports cells with the energy needed for life (Bräsen et al., 2014). In the present study, the new Parvarchaeota could potentially generate ATP via Embden-Meyerhof-Parnas (EMP) glycolysis pathway as most genes involved in this pathway were detected, including archaeal type glucose-6-phosphate isomerase, fructose-bisphosphate aldolase, triosephosphate isomerase, glyceraldehyde-3-phosphate dehydrogenase, phosphoglycerate mutase (or 2,3-bisphosphoglycerate-independent phosphoglycerate mutase), enolase and pyruvate kinase. However, the absence of *pfk* gene that is commonly found in other Parvarchaeota MAGs (Chen et al., 2018), which phosphorylates fructose 6-phosphate, suggests that they may employ an alternative pathway (e.g., degrading glycerone phosphate via the core module involving three-carbon compounds in glycolytic pathway) or alternative genes for *pfk*. Although genes involved in glycogen synthesis were not all presented in MAGs, the common detection of glycogen synthase implies that carbohydrate might be stored in some cases, which enables organisms to be advantageous when carbon sources are scarce (Santiago-Martínez et al., 2016). Due to the common detection of *korAB* genes, these MAGs might convert pyruvate to acetyl-CoA, which serves as a pivotal intermediate in core metabolism pathways. For instance, acetate could be generated by acetyl-CoA via acetate-CoA ligase, which the gene *acdA* encoding this enzyme was detected in genomes of this new family. Besides glycolysis, they could potentially generate pyruvate from D-lactate and malate because of the occurrence of *ldhA* (D-lactate dehydrogenase) genes and *maeA* genes (malate dehydrogenase), stressing the importance of pyruvate in metabolisms of this novel archaeal family. Considering that many genes in central carbon metabolism were present, we proposed that members of this family could obtain energy from these pathways to survive. However, further cultivation-based studies are needed to resolve the physiology of these archaea.

Although *petB* gene encoding for the cytochrome b subunit of ubiquinol-cytochrome c reductase was detected in 4 MAGs, the absence of iron-sulfur and cytochrome c1 subunit indicated that the complex III might not be present. Combined with the lack of complex IV where oxygen is ultimately oxidized, we proposed that this lineage adopts an anaerobic lifestyle. Notably, no MAG contains a complete V-type H^+^-transporting ATP synthase that includes nine subunits, and two MAGs (FK_bins.410 and TL1-5_bins.178) even do not contain any subunit of such ATP synthase. Such difference may be attributed to that these newly constructed MAGs showed a relative low completeness and these genes tend to be present or absent simultaneously as all genes associated with ATP synthase were in a small region (6–9 genes) in the MAGs.

Besides, only genes involved in nucleotides salvage pathways were encoded, like genes encoding for IMP dehydrogenase, nucleoside-diphosphate kinase, 5’nucleotidase, CTP synthase, dCTP deaminase, and so on. They might acquire nucleotides by nucleotides salvage pathways instead of *de novo* biosynthesis of nucleotides, albeit free nucleotides may be unstable in acidic environments. Notably, they may depend on an external source of lipids and vitamins because lipids and vitamin biosynthesis pathways were absent in all five MAGs. Combined with a limited set of metabolic repertoires, we proposed that they depended on partner organisms for growth. However, proteins involved in cell-cell interactions were limited in these MAGs, which only included two proteins in pili formation (virB11 and TadC). It remained to be determined whether this was a sign of genome streamlining or attributed to genome incompleteness. Further studies are needed to know how these MAGs interacted with other organisms for growth.

Several genes related to the SUF system were reported in these MAGs, including *sufB*, *sufC*, and *sufD*, encoded for Fe-S cluster assembly protein SufB, Fe-S cluster assembly ATP-binding protein, and Fe-S cluster assembly protein SufD, respectively. SUF system plays a pivotal role in Fe-S cluster assembly and sulfur assimilation (Zheng et al., 1993), suggesting that they may rely on sulfur compounds in the environments. Notably, the gene coding for the sulfocyanin, one of the blue copper-containing protein which plays a crucial role in iron oxidation in some archaea and bacteria lineages (Dopson et al., 2005; Castelle et al., 2015), has been found in four MAGs (FK_bins.410, MAS1_bins.189, MAS2_bins.147, and MAS3_bins.60), suggesting the involvement in iron oxidation. Phylogenetic analysis with high bootstrap values showed that these protein sequences are located in archaeal clades, and the closest relatives are *Ca. Mancarchaeum acidiphilum* and *Metallosphaera yellowstonensis* (Supplementary Figure 5). However, because they were quite different from other sulfocyanin proteins (Supplementary Figure 5), whether they could functions as other sulfocyanin sequences require further experimental confirmation.

### Environmental Adaptations

Characterized by low pH value and high concentration of dissolved metals, AMD represents an extreme environment for organisms, where microbes must cope with environmental stresses, including heavy metal, oxidative, and acid stress. For metal stress, two kinds of metal transporters were detected, including *copA* (3 MAGs) gene encoded for P-type Cu + transporter and *kch* gene (5 MAGs) that encode for voltage-gated potassium channel, revealing that they could resist against metal stress by efflux of metal ions. The detection of *arsC* gene (1 MAG) encoding for arsenate reductase, *merA* gene (1 MAG) encoding for mercuric reductase, and *chrR* gene (2 MAGs) encoding for chromate reductase enabled these MAGs to reduce metal ions or metalloids to less toxic reduced forms and then export them. In response to oxidative stress, all MAGs carry a thioredoxin reductase and a peroxiredoxin, which could help them defend against oxidative damage by reducing thioredoxin and regulating hydrogen peroxide levels. The occurrences of *ahpC* gene encoding for peroxiredoxin (alkyl hydroperoxide reductase subunit C), the *sodA* gene encoding for superoxide dismutase, and the *trxA* gene encoding for thioredoxin in some MAGs indicated that they could cope well with oxidative stress in many ways.

Also, many genes related to other environmental stress were detected in these MAGs. For instance, the *htpX* gene encoded heat shock protein HtpX, which is known for its role in heat stress. However, heat shock protein may also be involved in cold and UV stress (Matz et al., 1995; Cao et al., 1999). Besides the role in sulfur assimilation, the detection of the *sufB* gene in all MAGS might help them resist iron limitation and oxidative stress (Huet et al., 2005). The occurrence of the *fnr* gene encoding for ferredoxin/flavodoxin-NADP^+^ reductase suggested that all MAGs could protect cell against ROS-dependent cellular damage (Girardini et al., 2002). Gene *lhr* encoding for ATP-dependent helicase Lhr and Lhr-like helicase were found in all MAGs and could aid them in DNA repair when organisms are under UV irradiation or mitomycin C (Rand et al., 2003; Boshoff et al., 2003). Three MAGs (FK_bins.410, MAS3_bins.60, and TL1-5_bins.178) carry DNA repair protein SbcC/Rad50 and DNA repair protein SbcD/Mre11, which could prevent them from DNA damage caused by gamma radiation (Quaiser et al., 2008). In short, all the above findings have revealed the survival strategies of this lineage in AMD environments.

### Evolutionary History of Parvarchaeota

**FIGURE 5:**
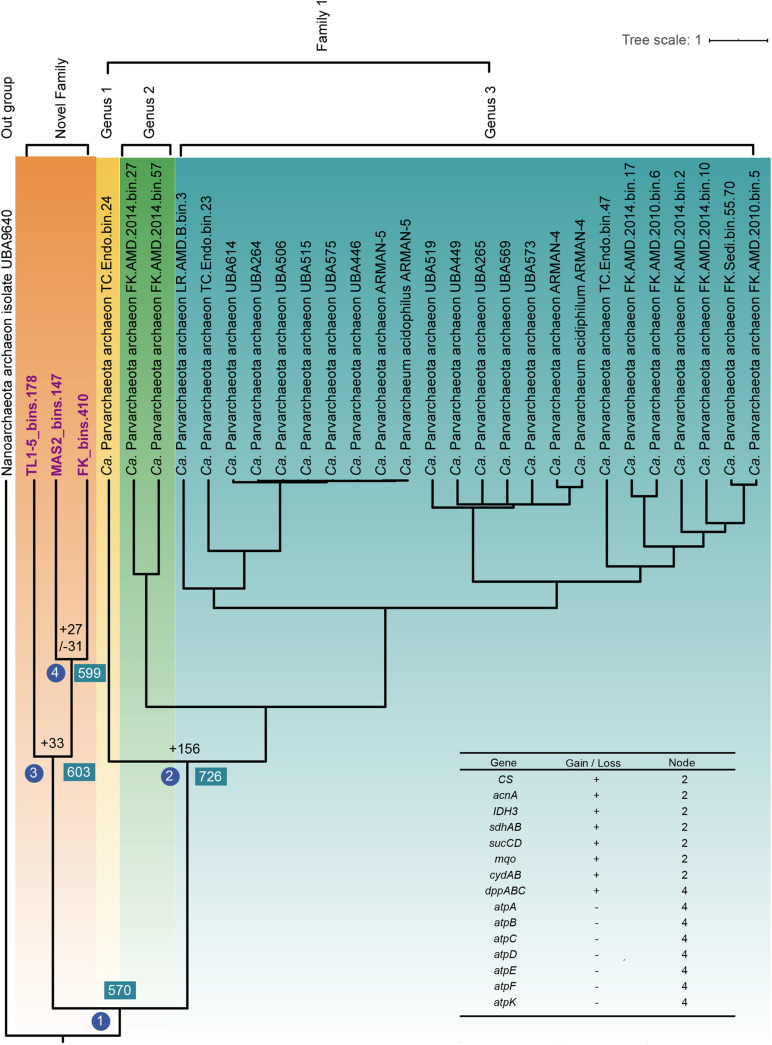
Ancestral gene family reconstruction inferred from the global gene family of all available Parvarchaeota MAGs (completeness ≥70%). The Bayesian tree is inferred from MrBayes (Ronquist et al., 2012). The number of gene gain (“+”) and loss (“−”) events were showed at the specific nodes of the tree. A list of essential genes gain or loss events mentioned were shown.

To reveal the evolutionary history of Parvarchaeota, gene gain and gene loss events were evaluated by mapping the OGs profiles to the Bayesian tree that was based on concatenated alignments of 122 archaeal-specific marker genes with high posterior probability (>97%, Figure 5). In total, 570 OGs were detected in node 1, which several of them could be classified as resistance-related genes, including *sbcCD*, *trxA*, *lhr*, *trxB*, *sodA, bcp, chrR, merA, arsC, aphC, grpE, dnaK, dnaJ*, and *sufB*, suggesting that the last common ancestor (LACA) of Parvarchaeota could cope well with environmental stresses. In the following, we will focus on the nodes of separation of two families and the formation of the novel family. At node 2, many gene gain events occurred, and some of them were metabolism-related. For instance, some genes involved in the tricarboxylic acid (TCA) cycle were acquired, including citrate synthase, aconitate hydratase, isocitrate dehydrogenase, succinyl-CoA synthetase, malate dehydrogenase, and succinate dehydrogenase/fumarate reductase, which could support microorganisms with key metabolic intermediates connecting carbohydrate, fat and protein metabolism. Besides, genes *cydAB* (cytochrome bd ubiquinol oxidase) and *sdhAB* (succinate dehydrogenase/fumarate reductase) might enable some of the Parvarchaeota members to respire oxygen in some way. At node 3, 33 gene gain events occurred, which lead to separations of the two families. Among them, the gene coding for PmbA protein was acquired and only presented in this novel family, which is involved in the toxin–antitoxin system (Pandey and Gerdes, 2005), suggesting that this system might play a role in the survival of members of this novel family. Besides, genes related to archaeal-type H^+^-ATPase were lost at node 4, indicating that some novel family members might generate ATP via alternative ways. Genes involved in an ABC-type dipeptide/oligopeptide/nickel transport system were gained at the same node, suggesting that they acquired the ability for peptides utilization by transporting them into cells and then degrade them.

In sum, different events occurred during the formations of two families, which enable them to possess the ability to utilize different substrates for survival.

## Conclusion

The new Parvarchaeota MAGs recovered from the AMD sediments have expanded the phylogenetic diversity of this little known archaeal phylum. Due to the inability to biosynthesize common amino acids and nucleotides, these uncultivated, ultra-small archaea may adopt a lifestyle depending on other community members. The predicted capacities for carbohydrates and protein utilization and environmental adaptation, including resistance to metal and oxidative stress, may enable these microbes to survive in the harsh environmental conditions. The detection of sulfocyanin suggests iron oxidation as a crucial energy-yielding pathway. Evolutionary history analysis showed that separation of the two Parvarchaeota families, represented respectively by the MAGs retrieved in the current study and those reported previously, led to the diversified acquired ability to utilize different substrates, allowing them to occupy different niches. This study contributes to a better understanding of the metabolic potentials and environmental adaptation of small-genome archaea in extreme environments.

## Data Availability Statement

The datasets presented in this study can be found in online repositories. The genome sequences of MAGs FK_bins.410, MAS1_bins.189, MAS2_bins.147, MAS3_bins.60, and TL1-5_bins.178 have been submitted to GenBank under the accession numbers GCA_015121965.1, GCA_015122035.1, GCA_015122065.1, GCA_015122075.1, and GCA_015122025.1, respectively. The names of the repository/repositories and accession number(s) can be found in the article/Supplementary Material.

## Author Contributions

Z-HL, QL, BL, and L-nH wrote the manuscript. Z-HL, QL, YL, and HC performed the metagenomic analysis, genome binning, functional annotation, and evolutionary analysis. LH guided the project. All authors discussed the results and commented on the manuscript.

## Conflict of Interest

The authors declare that the research was conducted in the absence of any commercial or financial relationships that could be construed as a potential conflict of interest.

## Footnotes

1 https://www.genome.jp/kegg/kegg1b.html

2 http://eddvlab.org/software/ssu-align/

3 http://www.arb-silva.de
