# Supplementary material for: Diversity and Genomic Characterization of a Novel Parvarchaeota Family in Acid Mine Drainage Sediments
Source: Front Microbiol. 2020 Dec 21;11:612257. doi: 10.3389/fmicb.2020.612257 (PMC7779479; doi:10.3389/fmicb.2020.612257)
Supplement: Supplementary file 2 [file Data_Sheet_2.docx]

**Genomic characterization of a novel Parvarchaeota family in acid mine drainage sediments**

Zhen-Hao Luo^1†^, Qi Li^1†^, Yan Lai^1^, Hao Chen^1^, Bin Liao^1^, Li-nan Huang^1*^

^1^School of Life Sciences, Sun Yat-Sen University, Guangzhou, 510275, PR China

^†^ These authors contributed equally to this work.

^*^ Correspondence author: L.N.H., eseshln@mail.sysu.edu.cn


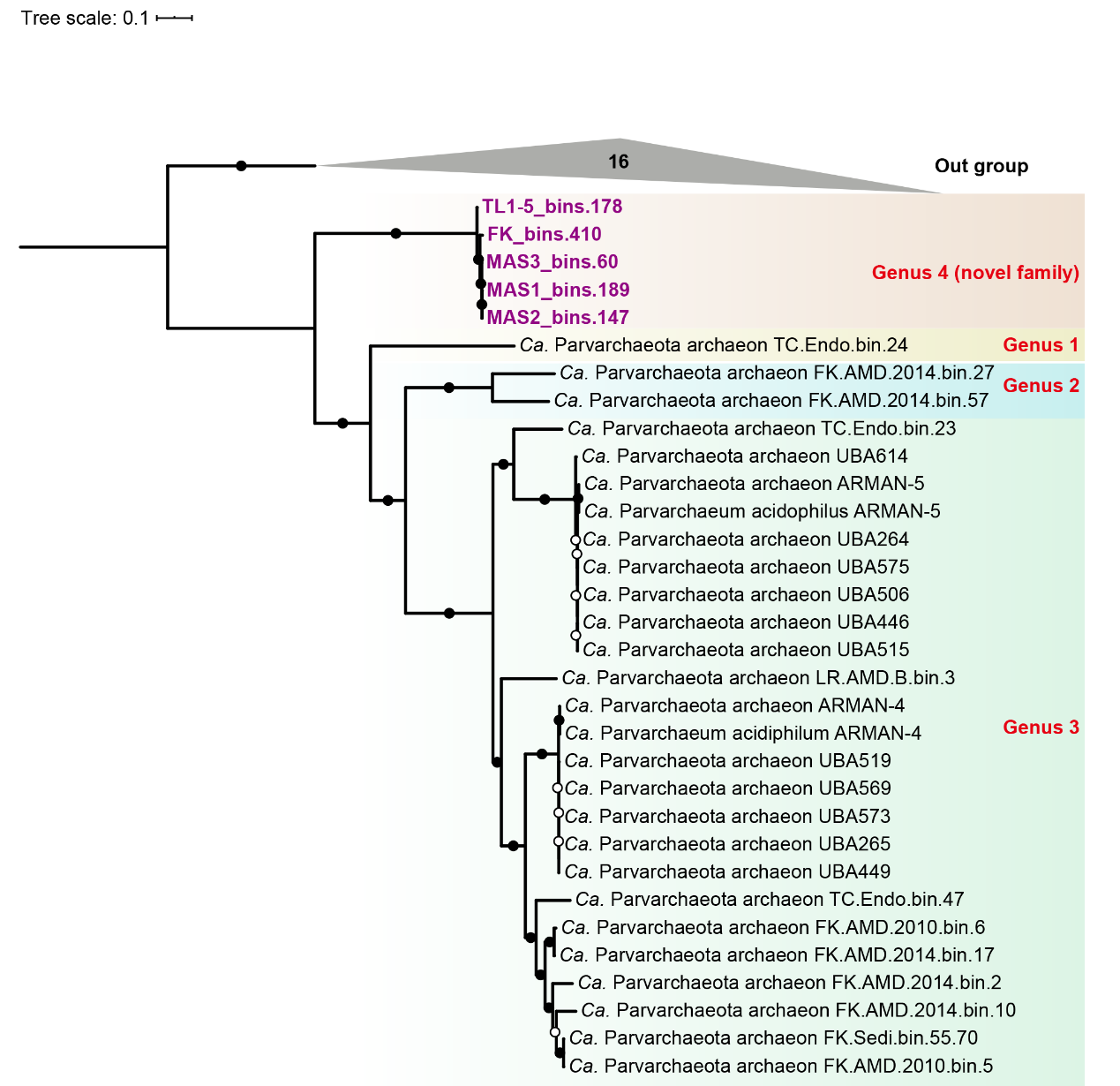


**FIGURE S1|** Phylogenetic tree based on concatenated alignments of 122 archaeal marker genes. Different clades are showed by boxes with diverse colours and MAGs newly obtained in this study are labelled with purple. Nodes with ultrafast boot strap ≥ 95% (50%) are indicated as solid (hollow) circles and the scale bar indicates 10% sequence divergence.


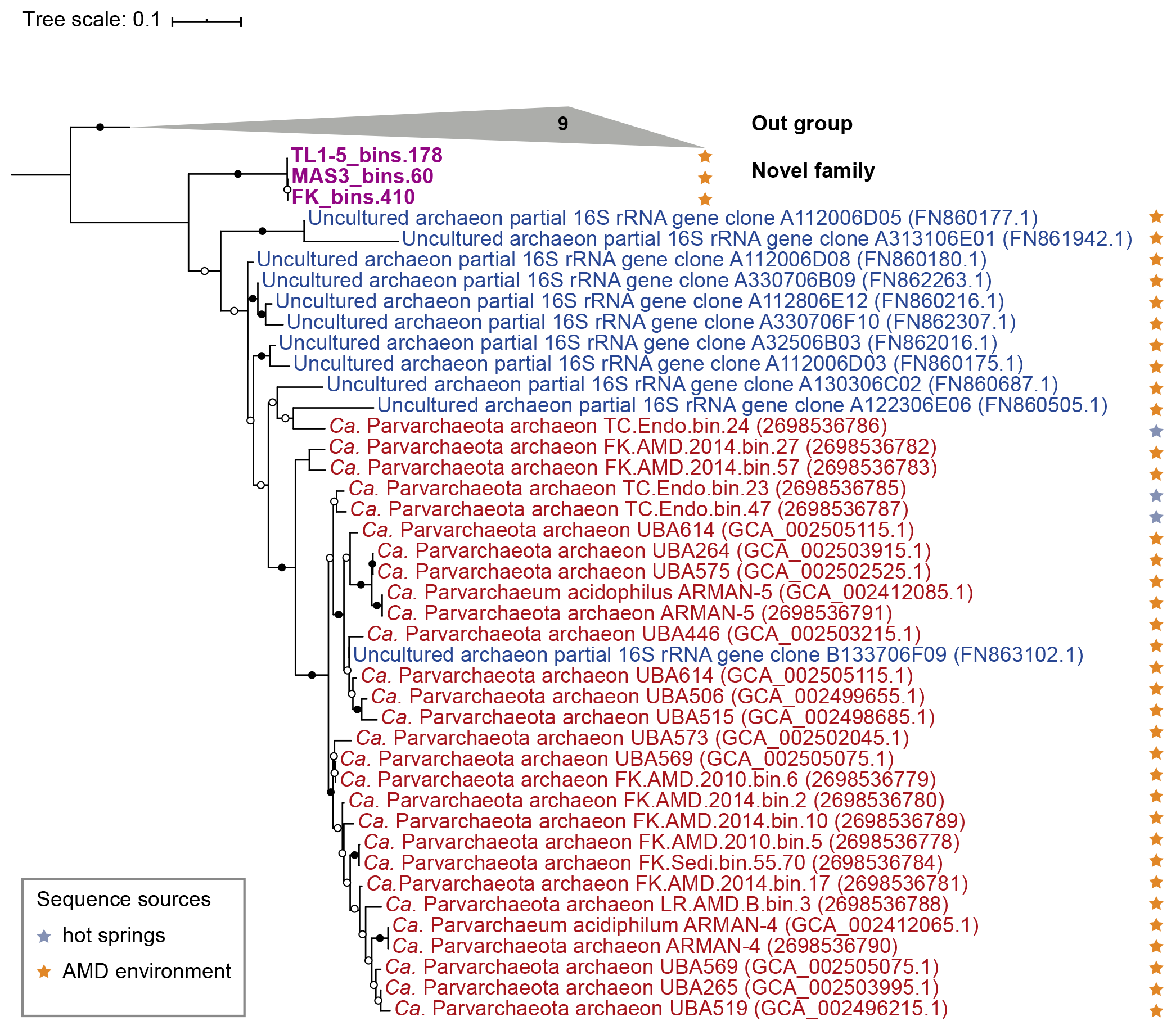


**FIGURE S2|** Phylogenetic tree based on 16S rRNA gene sequences from Parvarchaeota. Sequence sources of published 16S rRNA genes are indicated stars with different colors. Sequences with available published MAGs are labelled with red, without published MAGs are with blue and sequences from this study are with purple. Nodes with ultrafast boot strap ≥ 95% (50%) are indicated as solid (hollow) circles and the scale bar indicates 10% sequence divergence.


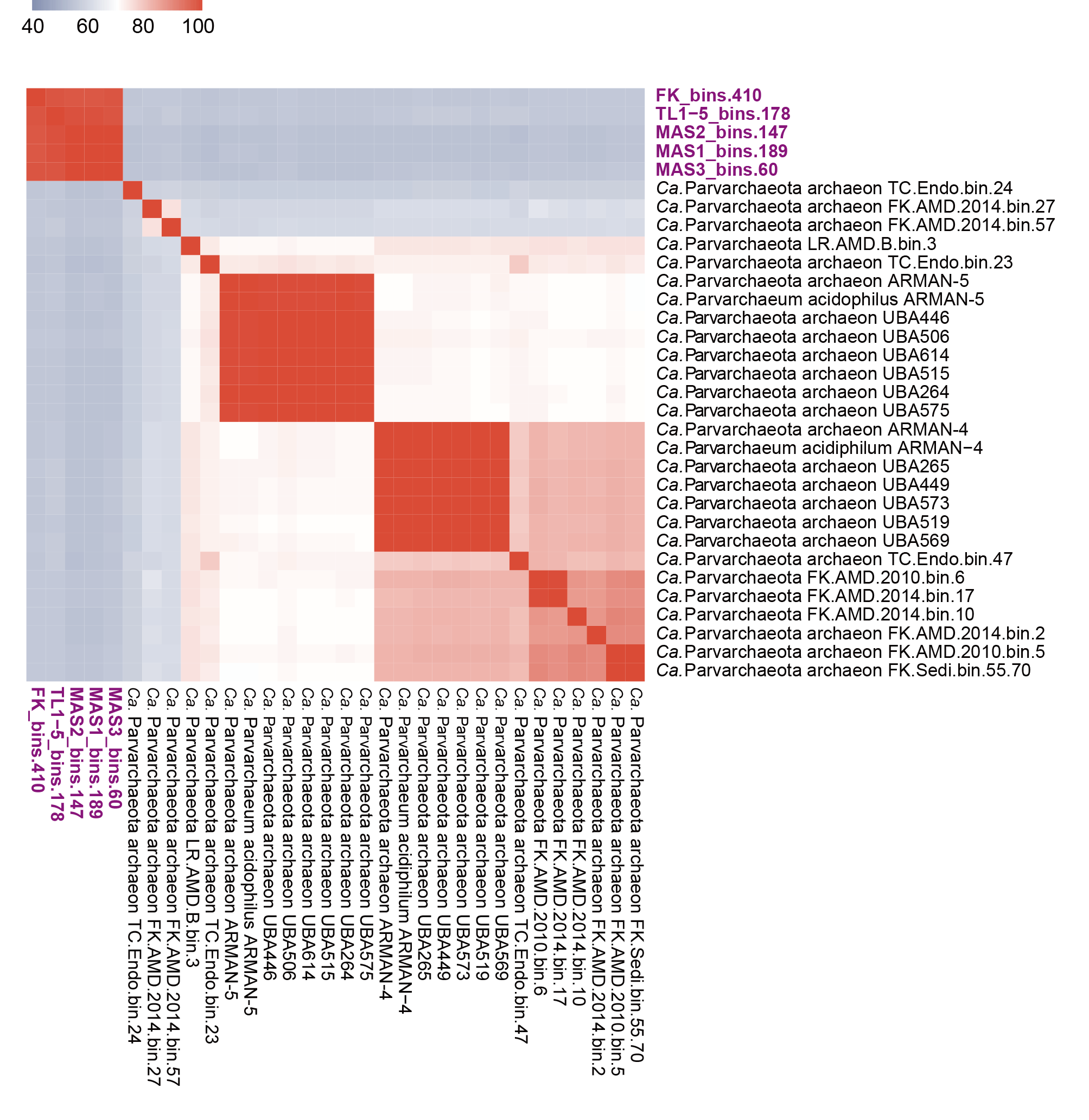


**FIGURE S3|** Average amino acid identity of all available Parvarchaeota MAGs. MAGs newly obtained in this study are labelled with purple.


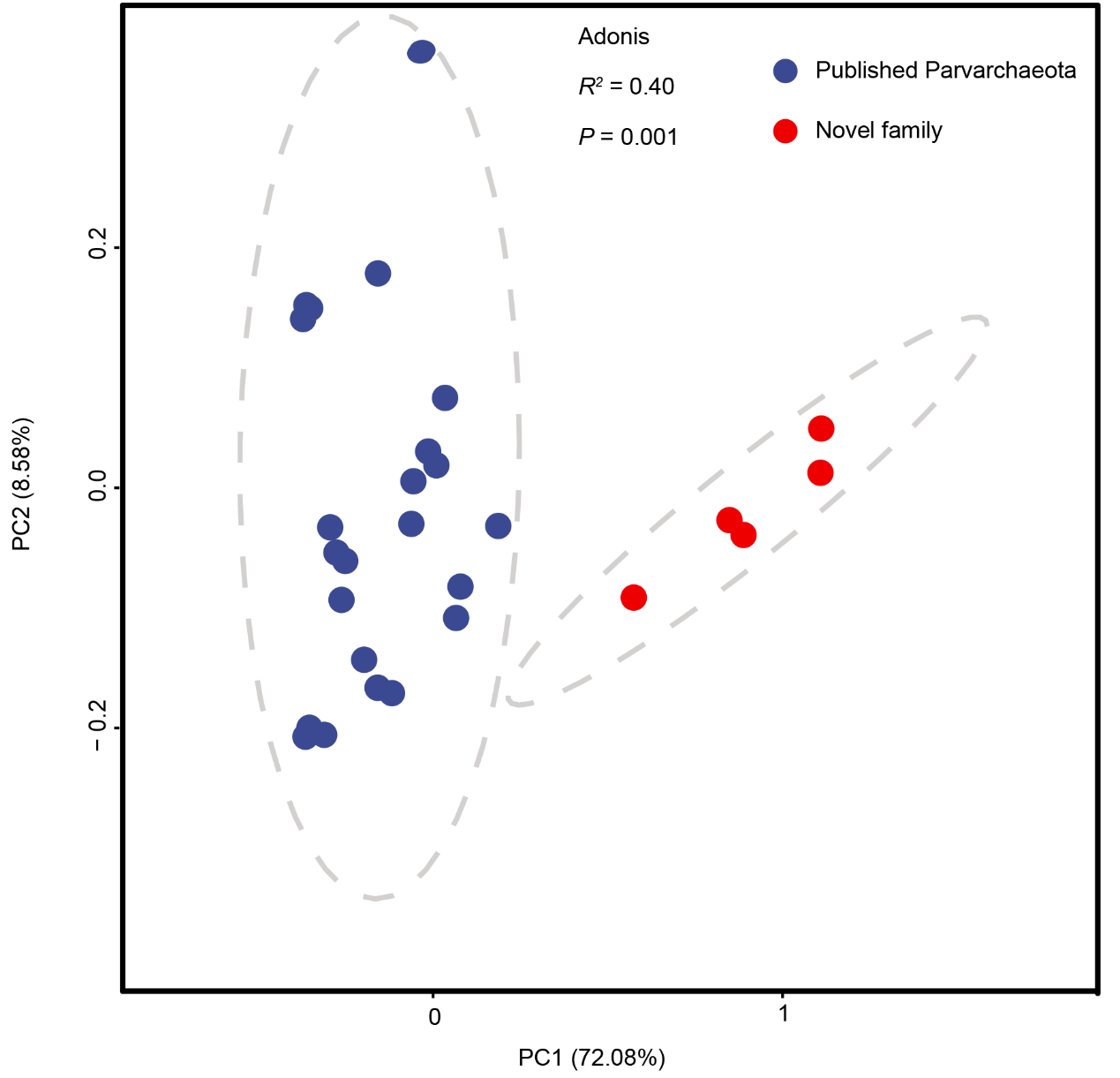


**FIGURE S4|** Principal coordination analysis (PCoA) plot with Bray-Curtis dissimilatory based on KEGG profiles of all Parvarchaeota MAGs. The analysis of similarity (ADONIS) statistics was based on comparisons between published MAGs and those obtained in this study.

**
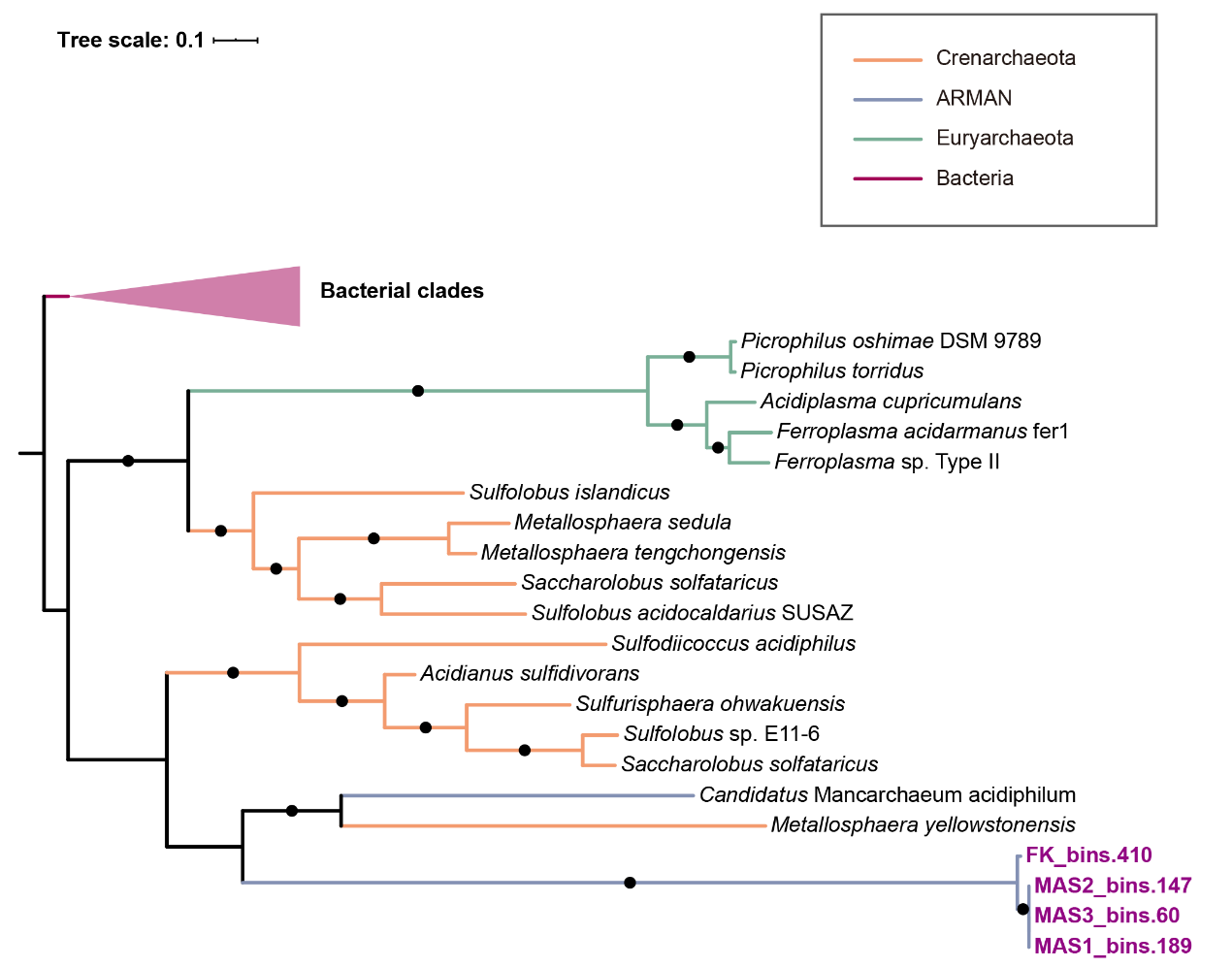
FIGURE S5|** Phylogenetic reconstruction of sulfocyanin. Sources of sequences were indicated in different colors and sequences from this study were marked with purple. Nodes with ultrafast bootstrap values ≥ 50% were marked with black dots, and the scale bar at the middle indicates 10% sequence divergence.
